# “You understand that whole big situation they’re in”: interpretative phenomenological analysis of peer-assisted learning

**DOI:** 10.1186/s12909-018-1291-2

**Published:** 2018-08-14

**Authors:** Shameena Tamachi, James A. Giles, Tim Dornan, Elspeth J. R. Hill

**Affiliations:** 1 School of Medical Sciences, The University of Manchester, Manchester, UK; 2 Washington University School of Medicine, Saint Louis, MO USA; 3 Centre for Medical Education, Queen’s University Belfast, Belfast, Northern Ireland UK

**Keywords:** Peer-assisted learning, PAL, Phenomenology, IPA

## Abstract

**Background:**

Peer-assisted learning (PAL) increasingly features within medical school curricula. While there is evidence of its effectiveness, less is known about *how* it promotes learning. Cognitive and social congruence between peer-tutor and student have been described as important concepts underpinning teaching and learning in PAL. We employed interpretative phenomenological analysis for an in-depth exploration of how medical students experience PAL sessions.

**Methods:**

We conducted the study at The University of Manchester within a near-peer scheme aimed at developing clinical skills within clinical clerkship students. We conducted individual interviews with three peer tutors and five students. We undertook interpretive phenomenological analysis of interview transcripts. We subsequently synthesised an account of the study participants’ lived experiences of PAL sessions from individual personal accounts to explore how medical students experience peer-assisted learning. This analysis was then used to complement and critique a priori educational theory regarding the mechanisms underlying PAL.

**Results:**

Students experienced PAL sessions as a safe and egalitarian environment, which shaped the type and style of learning that took place. This was facilitated by close relationships with peer-tutors, with whom they shared a strong sense of camaraderie and shared purpose. Peer-tutors felt able to understand their students’ wider sociocultural context, which was the most important factor underpinning both the PAL environment and tutor-student relationship. Participants contrasted this relative safety, camaraderie and shared purpose of PAL with teaching led by more senior tutors in clinical settings.

**Conclusions:**

This study provides a rich description of the important factors that characterise medical students’ experiences of PAL sessions. Participants felt a strong sense of support in PAL sessions that took into account their wider sociocultural context. Multiple factors interplayed to create a learning environment and tutor-student relationship that existed in contrast to teaching led by more senior, clinical tutors. The insight generated via IPA complemented existing theory and raised new lines of enquiry to better understand how the peer relationship fosters learning in PAL at medical school. We make recommendations to use insights from PAL for faculty and curriculum development.

**Electronic supplementary material:**

The online version of this article (10.1186/s12909-018-1291-2) contains supplementary material, which is available to authorized users.

## Background

Peer-assisted learning (PAL), defined as “the acquisition of knowledge and skill through active helping and supporting among status equals or matched companions”, [1] has been in formal operation in higher education in the UK since 1990 [2]. Within undergraduate medical education, the provision of peer-led, cooperative study sessions supplementing traditional teaching has become a valued teaching method over the past three decades [3, 4].

Numerous quantitative studies have sought to evaluate the effectiveness of PAL by examining the academic performance of students tutored by their peers. Several researchers have observed comparable test scores in groups of individuals offered PAL sessions within their usual medical curricula, in addition to benefits beyond medical knowledge [5–8]. Although such findings provide impetus for medical schools to develop their own versions of PAL, of greater theoretical interest to medical educators is the issue of *how* the interactions between peer-tutors and students foster learning.

Lockspeiser et al. have highlighted the value of cognitive and social congruence in one medical school’s supplemental peer-teaching programme [9]. Cognitive congruence reflects the ability of tutors to engage with students at an appropriate level, drawn from a shared understanding of the material under discussion [10]. Social congruence has been described as a willingness to become involved with students in an authentic way [10, 11]. In terms of how such phenomena foster learning, Ten Cate and Durning, in their 2007 review paper, clarify the benefits of peer learning for students, which are also based in cognitive and social congruence [10]. Similar themes of empathic, supportive relationships as a basis for learning in PAL sessions were highlighted by Tai et al. in their systematic review of same-level PAL schemes [8]. Tai and her colleagues identified further benefits of PAL within the medical education literature including professional identity formation. Much of the PAL literature reports the structure of schemes and their outcomes in terms of post-intervention test scores. There has been relatively little focus on developing a qualitative understanding of the nature of learning within PAL [12]. Further, the literature fails to explain the disproportionate popularity of PAL among medical students, despite equivocal evidence for its effectiveness [8]. Hence, a richer understanding of the medical student *experience* of PAL sessions may help to further develop its underlying educational theory, both in terms of elucidating further the known mechanisms and in uncovering new ones.

Over recent years, there has been a call to gain deeper insight into students’ experiences of medical school. In this paper, we use interpretative phenomenological analysis (IPA) as our methodology. IPA is ideal when seeking to understand participants’ lived experience of a context or phenomenon, and relatively well-established in the medical education literature. In a review of qualitative methodologies in medical education, Cleland cites two exemplars of IPA [13]. These papers demonstrate how IPA can help us gain insight into medical students’ experiences and thus develop theory as to *how* learning happens – in these examples, the authors seek to understand the experience of learning while on placement at nursing homes, [14] and to understand how students make meaning from experiences of empathy [15]. In both cases, the authors use their phenomenological analysis to inform and better ground a priori educational theory in the lived experiences of students. In this IPA study, we seek to examine the specific research questions: how do medical students experience the peer relationship within PAL sessions, and how does this experience mediate learning?

## Methods

In this report, students who act as peer-tutors are referred to as *tutors*, and the learners in PAL sessions as *students*.

### Setting

Ethics approval was obtained from the UK National Research Ethics Service (North West – Preston Committee, REC reference number 11/NW/0311). The study was conducted at a teaching hospital (Salford Royal NHS Foundation Trust, SRFT) within Manchester Medical School. The degree programme is a 5-year problem-based learning course, the final 3 years of which are spent on clinical placement. The PAL scheme at SRFT runs sessions for over 250 medical students each academic year. Interactive seminars covering clinical topics – peripheral vascular examination, neck examination, fluids management, and x-ray interpretation – are led by Year 4 students and offered to students in Years 3 to 5 – a near-peer scheme. It is voluntary for students to attend and the sessions supplement the established curriculum. Consultant (attending) doctors are invited to review teaching content for accuracy, though they do not attend sessions. Teaching is delivered by pairs of tutors to 12–20 students. To apply to be a tutor, applicants must complete electronic applications comprising open-ended questions on relevant experience and motivations for the role. Tutor selection occurs via annual recruitment overseen by existing tutors.

### Participants

Study information was sent by email to all students at SRFT who had either taught or attended at least one PAL session in the 2010–2011 academic year. We recruited three tutors via email advertisement from a pool of twelve, and five learners for this study from a pool approximately twenty who responded. Thus, we selected a sample of different year groups including both those who entered medical school directly from secondary school and mature students. This allowed comparison between different perspectives within the single context of PAL sessions. We obtained written informed consent from all participants. Interviews were conducted within 6 weeks of a PAL session, to ensure participants had recent recollections. After the interviews, they were offered certificates of participation for their portfolios. Participants were interviewed by a fellow student, were told their data would be confidential and would neither be available to the medical school nor influence their grades.

### Procedure

Given our focus on individual students’ lived experiences in PAL sessions and the need to maintain confidentiality, we conducted semi-structured interviews in private surroundings, such as a private study room. ST conducted all interviews, which lasted for 30–60 min. Participants were asked about their experiences in PAL sessions and clinical teaching, the roles of the PAL tutors, and their reasons for participation in PAL. As the aim of the interview was to obtain an account of participants’ experiences, the interviewer exercised as loose control over the discussion as possible, often departing from the interview template (Additional file 1). Interviews were audio recorded, and transcribed verbatim for analysis. Pseudonyms were assigned to protect participants’ identities.

### Analysis

Phenomenology is the study of human experience and the way things are perceived as they appear to consciousness [16]; it is concerned with lived experience. From a phenomenological perspective, different people can, and do, live through the same environment in radically different ways [17]. Interpretative phenomenological analysis (IPA) enables research of individuals’ experiences [18], where IPA is idiographic, uses purposive sampling, and goes beyond purely descriptive approaches to uncover the meaning of the experiences of a specific group on a specific topic [16, 19]. IPA deliberately uses small, homogenous samples of respondents to gather detailed information about their experiences [17]; typically, 10 or fewer respondents [20]. This is in contrast to descriptive phenomenology, where maximum variation sampling is common. Hence, while our participant selection included a variety of medical school years and educational backgrounds, the participants all shared the single context of the SRFT PAL scheme.

With its emphasis on the detailed exploration of participants’ viewpoints, the method makes limited claims to generalisability and draws conclusions via rigorous thematic analysis. Rather than assuming a priori to understand how the peer relationship may foster learning in PAL sessions, IPA aims to be responsive to the voice of each research participant and to adopt an analysis that represents ‘an insider’s view’ [21, 22]. It subsequently incorporates the insight generated into existing theory [19].

In keeping with established IPA method, rather than setting out with an a priori theory of how students experience PAL sessions, we first constructed an initial set of themes from recurrent reading of four participants’ narratives [19, 21–23]. These were subsequently grouped into wider themes representing a broader analysis. Phenomenological reduction was undertaken via identification of the constituents of students’ experiences within PAL interactions [24]. On reading of the remaining transcripts, we modified existing themes. Variations in different researchers’ interpretations were resolved by discussion of higher order themes. The template of hierarchically arranged themes that emerged was used to examine the data to answer the research questions, with the focus being on consistencies in participants’ accounts of particular issues [19].

Recognising that the involvement of an interested researcher as an active participant influences the processes and the knowledge produced by research [17], here we describe briefly the backgrounds of the researchers and how we addressed reflexivity. ST at the time was a fourth year medical student and past peer-tutor embarking on a medical education project option. JG and EH were both MB PhD students (neuroscience and medical education respectively) and past peer-tutors. TD was Professor of Medicine and Clinical Education at the teaching hospital at which the PAL scheme was based. ST, who conducted the interviews, wrote an inventory of preconceptions about the study at the start, and maintained a research diary to record reflections about each interview. Known as *bracketing* – setting aside what is thought to be known about the phenomenon under investigation [20] – this was the means through which the team achieved reflexivity, and raised awareness of any biases which were brought to the analysis. TD conducted a validation check, comparing the final themes with the transcripts, to confirm representativeness [18, 19].

## Results

Safety and egalitarianism were linked with the learning environment in PAL sessions. Both students and tutors experienced sessions as “very relaxed and informal”. The PAL learning environment was experienced as different, in this particular way, from that of teaching led by faculty in clinical settings. This difference was not related to the form the teaching took; participants described slide presentations and “small group practice” occurring in both ‘types’ of teaching session. More, it was a sense of being comfortable in the PAL environment that mediated students’ understanding of their role in sessions.

Here, Ben describes what we came to understand as the ‘threat of the ward’ - a sense of vulnerability experienced by medical students (tutors and students alike) during clinical teaching with faculty.

…there’s also a lot of stories of teaching by humiliation and singling out and pressure to get things right, and... being afraid of... getting things wrong. So for me, they [clinical settings] weren’t always the most useful environments in which to learn, and certainly I would not volunteer, which would help my learning, but be afraid of being wrong or embarrassment would be so bad.

Ben, tutor

As Robert delineates below, the anxiety provoked by clinical learning environments framed his experience of the relative safety of PAL sessions. This is important as participants directly related their ability to learn to the safety of their environment.

...it’s from having... a safe environment to explore [a topic]... it goes back to… the informal set up of PAL in the sense that on a ward round with a consultant [attending physician], you’re less likely to ask questions. There’s always that fear you could get shot down and feel stupid.

Robert, tutor

While the experience of developing one’s knowledge without the fear of embarrassment or even humiliation was important, there was also a strong sense of feeling more self-confident in PAL sessions. Here, Scarlett expresses how feeling uncomfortable in her own skin relates directly to her experience of learning.

If you don’t have a safe learning environment, if you’re not comfortable, you’re not going to be taking as much in.

Scarlett, student

Egalitarianism was prominent in students’ experience of the environment; they felt legitimacy in their roles as session participants, which led to feeling free to participate more fully in sessions. They also reported ‘closeness’ between tutors and students that appeared to flatten the hierarchy implicit in tutors’ social position as more senior medical students. In this way, the closeness changed their experience of the session environment.

“the tutors… tried to create an environment where everyone feels included in the discussions”.

Nadia, student

“So it’s like a close working group, rather than… it feels more like that than a teaching session.”

Ben, tutor

The experience of informality was also central to the relationship between students and tutors. Here, there was a strong experience of joint purpose shared by tutors and students in sessions. Again, the oppositional nature of ‘clinical teaching’ provided a context for this experience.

The idea of… a similar purpose in the sense that you’re still students… you have the same goal, which is to pass medical school. Camaraderie is probably sort of the kind of word… you’re on the same side you see. We’re all learning together. I know what you’re going through.

Bob, tutor

And that’s such a unique thing for peer assisted learning because we naturally have that camaraderie, and we want to support each other and see each other excel...

Melody, tutor

This sense of camaraderie led students to experience the formation of deeper, more lasting relationships even after the PAL sessions were complete.

I think there’s definitely sort of the aspect of sort of friendship in terms... because I know that some people are now friends with some of the past tutors. So I think there’s definitely that aspect to the relationship…

Scarlett, student

The intimacy of PAL sessions fostered a more personalised experience for students in comparison to the anonymity felt in a clinical setting as ‘just one of the medical students’.

They were not just the person doing this PAL session afterwards… they took time out of their day and they emailed me back, and what’s more, they attached some things. And so that all adds to it… and then you think, “You know what? She’s a really nice person.” When I see her next time… I’ll say “hi”. Whereas with a reg [Registrar - senior postgraduate trainee] that I see racing past me in the corridor, nine times out of ten I will not say “hi” because they don’t see me and they’re looking right past me and they probably don’t even remember me. So I think it’s much more personal, the PAL set up, as opposed to: they’re just medical students turning up on the ward for teaching.

Nadia, student

Central to the relationship was the ‘shared journey’ of medical school between students and tutors. This was important as it allowed tutors to act as mentors, offering direction beyond the explicit remit of PAL session. This was experienced in different ways by different participants – from direct advice on specific projects to a wider sense of how to navigate different aspects of medical school successfully. Hence, there was a strong sense of trajectory that facilitated shared understanding between tutor and student.

This was I think before the session that they gave, back in January, when I’d not really written an SSC [Special Study Module - essay project], and I came up to medical school when I was 18, so for me it was the first proper like written piece of work that I’d done, and I think she actually showed me an example of a case report that she’d done which gave me an idea about structure and layout and how to best approach that situation…that was not even just how to survive the curriculum but the specific curriculum at Manchester, just the Manchester tailored curriculum and the best ways to get through or what they found to be effective…

Charlotte, student

So how can I do this? How do I become an x-ray tutor? How do I get involved in PAL? Can you recommend me?... Does it look good on your CV?… I have been asked what I do in [fourth year] to get further… A lot of third years are anxious about… the demands of Year 4. And a lot of fourth years… are anxious about whether to intercalate or not.

Melody, tutor

Tutors also acted as mentors and enabled access to an insider’s perspective on how to be a clinical medical student.

They don’t ask like what’s going to come up and things like that but just how to answer, how to behave.

Nadia, student

The themes discussed above – the environment, the relationship, and the experience of learning – have in common one particular aspect that was integral to PAL: the experience of shared understanding between students and tutors. Ben articulates this well, and discusses how this understanding feeds into his role as a tutor.

[I was] trying to imagine what it was like being a third year. It was the first year of clinical, which is a big step… because it’s your identity as a medical student… Second of all, it’s quite scary at times, being on the ward and practising clinical skills. You feel, or I felt… that there’s so much to learn and there are very, very high-pressure examinations at the end of the course… What I mean by empathising is that you understand that whole big situation that they’re in because you’ve just done it or you are doing it.

Ben, tutor

## Discussion

Our interpretative phenomenological analysis of medical students’ experience of PAL sessions showed how students experienced PAL sessions as a safe and egalitarian environment, which shaped the type and style of learning that took place. Safe learning environments have been addressed frequently by educational researchers, and tutors are aware of the importance of establishing positive learning climates in which students may make mistakes [10]. The PAL environment was facilitated by close relationships with peer-tutors, with whom students shared a strong sense of camaraderie and purpose. Peer-tutors felt able to understand their students’ wider sociocultural context, which was the most important factor underpinning both the PAL environment and tutor-student relationship.

The concept of ‘social congruence’ – a willingness to become involved with students in an authentic way – has been empirically linked with effective PBL tutors and theoretically linked with PAL in medical education [10, 11]. Schmidt and Moust examined the relationship between tutors’ personal qualities and students’ learning in the context of problem-based learning (PBL) [11]. While testing their model of effective tutorship, they found that social congruence – the readiness of tutors to seek informal relationships with students, to display an attitude of personal interest and caring, and to convey genuine interest in students’ lives and their learning – facilitated constructive group functioning. Here, our IPA approach has provided richer detail of how this concept manifests in the experiences of medical students in PAL sessions, and the implications it has for the session environment, tutor-student relationship and learning itself.

Throughout the analysis PAL was contrasted with teaching led by more senior tutors in clinical settings. Given that the factor that distinguishes PAL from other modalities is that it is led by peers, it is perhaps unsurprising that PAL sessions are chiefly experienced relative to teaching sessions led by senior clinical tutors. This was, however, a comparison that recurred throughout each theme: the PAL environment, tutor-student relationship and even learning via PAL were experienced in relation to teaching by senior clinical tutors. This relative account gives rise to the idea that PAL exists ‘in dialogue’ with senior, clinical teaching. Ben alludes to this when he uses the word “stories” to relate the experience of senior clinical teaching. This point shows the power of IPA to illustrate how students make meaning from their teaching and learning experiences, and from PAL in particular, whilst at medical school. This idea may further be examined with discourse analysis: it may be unimportant for a student to actually experience “humiliation... singling out and pressure to get things right” – the existence of stories is enough for the ‘threat of the ward’ to exist, and for the construction of students’ understanding of PAL in response to it. It is also interesting that a recent activity systems analysis of PAL in a *clinical* setting described tension arising from a clash of activity systems – from taking a ‘safe’ PAL approach in the relatively ‘unsafe’ ward environment [25].

Another interesting component of the PAL experience was that of peer mentoring that exceeded the explicit remit of sessions. Nadia delineated this experience when she recounts learning “how to answer, how to behave.” A recent grounded theory analysis of medical students’ experience of surgery delineated how medical students uncover and enact the surgical hidden curriculum [26]. This paper uses Hafferty and Hafler’s concept of the hidden curriculum as “the culture, beliefs and behaviours enacted by those within a community and passed to students, who subsequently enact them themselves” [27]. Hill et al. make a call for strategies to make the hidden curriculum more explicit as a potential mechanism to enhance participation of students who may otherwise be excluded [26]. PAL may be an educational tool to deliver this. In our study, peer-tutors explicitly shared their understanding of the hidden curriculum – their practical know-how of how to be a clinical medical student. The idea of tutors as purveyors of the hidden curriculum fits well within sociocultural theory, notably theories of situated learning such as Communities of Practice, which has previously been applied to PAL [28–30]. Here, learning is recognised as occurring through collaboration with other learners and more senior community members in carrying out purposes connected explicitly with the history and current practices of the community [31]. Situated learning illustrates the students’ sense of their learning experiences in the PAL sessions: the feeling that they were gaining strategies for getting through medical school. Recently this has been reframed as ‘professional congruence’ with particular reference to PAL [32]. This understanding of trajectory fits with a developing understanding of paradigmatic trajectories as an essential component of situated learning [29, 33]. Here, the paradigmatic trajectories of navigating medical school are both openly discussed by peer-tutors, and imbue the experience of teaching and learning activities within PAL sessions.

Among this study’s limitations was that we did not revalidate the findings with participants due to logistical difficulty of reconvening participants. Conscious from the outset that this might be likely, our approach included a final review of the analysis by TD and so this was tempered by a degree of internal validation, yet methodologically this is not the same. In terms of critical reflexivity, phenomenology requires researchers to ‘bracket’ their preconceived ideas regarding the focus of study. While we adhered to this protocol, the interviewer’s position as a medical student ‘insider’ and former peer-tutor influenced the eventual analysis. As an interpretative phenomenological analysis, this study’s findings have limits on their generalisability beyond its context. Yet, the strength of this study lies in a rich account of how medical students experience PAL sessions, the meaning they construct – in terms of knowledge and relationships within PAL sessions, and of their trajectory within a broader professional context – and provides insights into how PAL contributes to clinical education. As discussed above, the analysis has raised important new lines of enquiry to determine how the peer relationship fosters learning at medical school.

In terms of implications, this study raises issues for both future research and practice. The concept of social congruence needs to be further explored and could be done so from a sociocultural perspective to better elucidate underlying mechanisms of how PAL fosters learning. Methodologically, this study highlights how IPA allows particular insights into how medical students make meaning from their experiences at medical school, and how this may be used to critique and complement the prior literature. For peer-tutors, an appreciation of the impact of camaraderie and shared experiences on teaching and learning may help them to cultivate more effective teaching and learning environments. Whilst the results will be of interest to those developing PAL schemes, social congruence may also be relevant to faculty development; there may be advantages in equipping faculty with tools to foster a greater degree of congruence with and between their students. The peer-relationship fosters a safe and egalitarian learning environment; PAL may also be utilized as part of wellbeing, personal development and medical humanities aspects of medical school curricula in the future.

## Conclusions

Students felt a strong sense of support in PAL sessions that took into account their wider sociocultural context, which facilitated their experiences of teaching and learning. Multiple factors interplayed to create a learning environment and tutor-student relationship that existed in contrast to teaching led by more senior tutors in clinical settings. The analysis raises new lines of enquiry to better understand how the peer relationship fosters learning in PAL at medical school.

## Additional file

Additional file 1: Interview Discussion Schedule. (PDF 309 kb)

## Abbreviations

EH: Elspeth Hill
IPA: Interpretative phenomenological analysis
JG: James Giles
PAL: Peer-assisted learning
SRFT: Salford Royal NHS Foundation Trust
ST: Shameena Tamachi
TD: Tim Dornan
