# Supplementary material for: “You understand that whole big situation they’re in”: interpretative phenomenological analysis of peer-assisted learning
Source: BMC Med Educ. 2018 Aug 14;18:197. doi: 10.1186/s12909-018-1291-2 (PMC6092812; doi:10.1186/s12909-018-1291-2)
Supplement: Supplementary file 1 — Interview Discussion Schedule. (PDF 309 kb) [file 12909_2018_1291_MOESM1_ESM.pdf]

1-to-1 Interview Discussion Schedule

How do medical students experience the PAL interaction?

Academic Year 2010-11

Our chosen methodology is interpretative phenomenology. This would aim to establish a description of the experiences of others in the most faithful way possible, rendering these into theoretical interpretation. The interaction between the researchers and participants produces the data. The researcher is aiming, therefore, to observe and define the experiences of the participant. While there should be a focus to the interview, basing it around attempting to describe a particular experience, the discussion itself should be led by the participant and not the researcher. Therefore the pre-established discussion schedule comprises overarching themes only, and not specific questions so as to avoid influencing the participant's description of the experience.

This schedule is a guide only – variation in discussion will be expected. The schedule is centred around key themes, based on the research questions. The discussion will begin with the open questions listed below, and the interviewer will exercise discretion in probing the participant for greater detail where appropriate.

The student researchers have had at least four years of interview training as part of their undergraduate medical course, and are therefore well-versed in technique and interview skills.

A. General

Describe your experiences in the PAL sessions.

B. Specific

Describe an experience interacting with one particular PAL tutor.

1. Environment

How did you experience the environment of the PAL session?

2. Relationship

Describe the relationship which existed between you and the PAL tutor.

3. Closeness

Could you compare that experience with the one you had with the PAL tutor? What were the main differences?

4. Roles

What function did you see the PAL tutor fulfilling in the PAL session you attended?

5. Motivation – *explore in interview with PAL tutor*

Why did you choose to become involved with PAL?

6. Learning

What did you learn? How did you learn it?

How did peer assistance contribute to your learning?

Was your learning experience in the PAL setting any different other teaching settings?

Has being in a PAL session made you aware of teaching techniques that work best for you?
